# Risk factor analysis for central nodal metastasis in papillary thyroid carcinoma

**DOI:** 10.3892/ol.2014.2667

**Published:** 2014-11-04

**Authors:** LING-NA MAO, PING WANG, ZHI-YU LI, YONG WANG, ZHENG-YA SONG

**Affiliations:** 1 International Health Care Center, The Second Affiliated Hospital of Zhejiang University School of Medicine, Hangzhou, Zhejiang 310009, P.R. China; 2 Department of General Surgery, The Second Affiliated Hospital of Zhejiang University School of Medicine, Hangzhou, Zhejiang 310009, P.R. China

**Keywords:** papillary thyroid carcinoma, central neck dissection, total thyroidectomy, hypoparathyroidism, vocal cord paralysis

## Abstract

Lymph node involvement is associated with recurrence in papillary thyroid carcinoma (PTC). The central neck compartment (level VI) lymph nodes are at the greatest risk of metastases from PTC, but the role of central neck dissection (CND) remains controversial, particularly in PTC without clinical cervical lymph node metastasis (cN_0_). The present study aimed to identify risk factors of central cervical nodal metastasis and the safety of CND in patients with cN_0_ PTC. The current study retrospectively investigated 389 patients who had been followed up for 12.0–25.5 months after surgery, and were divided into positive or negative lymph node involvement groups according to the pathological results subsequent to this surgery. Univariate and multivariate analyses were used to study the risk factor of central node involvement. The mean tumor size was 0.71±0.35 cm (range, 0.1–2.0 cm). There was no significant difference in the rate of central lymph node involvement based on age (<45 or ≥45 years) or tumor focality (unifocal or multifocal). However, there were significant differences based on gender, extra-thyroid invasion and tumor size (P<0.05). The incidence of transient hypoparathyroidism and transient vocal cord paralysis following CND was 12.34 and 4.11%, respectively. No patient experienced permanent hypoparathyroidism or vocal cord paralysis. One patient (1/389; 0.23%) experienced disease recurrence during the follow-up. A larger tumor size and the male gender were significantly associated with the central nodal metastasis rate for cN_0_ PTC with a tumor size of <2.0 cm. CND for cN_0_ PTC patients was safe and the tumor-associated recurrence rate following CND plus total thyroidectomy was low. The present study suggests that CND should be conducted for male cN_0_ PTC patients with a larger tumor size (≥0.5 cm).

## Introduction

Papillary thyroid carcinoma (PTC) accounts for >90% of newly diagnosed thyroid cancers. The incidence of PTC has been increasing in previous decades, without significantly modifying the characteristic low aggressive behavior, in spite of frequent lymph node involvement (1). Overall, 20–50% of patients with PTC experience cervical lymph node metastasis (2). Usually, metastases from PTC occur in a stepwise fashion from the central to lateral neck compartments. Therefore, the central compartment lymph nodes are at the greatest risk of metastases from PTC. While the effectiveness of therapeutic central neck dissection (CND) is undisputed, there is no consensus on the role of CND in clinically node-negative patients with PTC. Proponents of CND propose that CND offers more accurate staging and may decrease the probability of locoregional recurrence (2–4). Opponents of elective CND highlight the potential for increased morbidity secondary to the risk of recurrent laryngeal nerve injury and hypoparathyroidism (5,6). The overall risk and benefits of the CND procedure must be determined on a patient-by-patient basis. Therefore, the purpose of the present study was to analyze the safety of CND and the risk factors of central nodal metastases in PTC without clinical cervical lymph node metastasis (cN_0_). Written informed consent was obtained from all patients.

## Patients and methods

### Patients

The present study was a three-year single institutional retrospective study, approved by the Medical Ethics Committee at The Second Affiliated Hospital of Zhejiang University School of Medicine (Hangzhou, Zhejiang, China). The inclusion criteria of the study were as follows: i) Pathologically confirmed PTC; ii) pre-operative ultrasonography, with or without computed tomography (CT) imaging of the thyroid and cervical lymph node basins; iii) no evidence of nodal disease based on negative physical examination, negative findings on pre-operative neck ultrasonography or CT, or the presence of lyphadenopathy <5 mm in diameter (cN_0_) (7); iv) normal vocal cords, confirmed by flexible fiberoptic laryngoscopy, and a normal parathyroid hormone (PTH) level (range, 15–65 pg/ml); v) CND (ipsilateral or bilateral) during planned total thyroidectomy (primary or completion); and vi) medical records and histological data available for review. A total of 389 patients who underwent CND for PTC between January 1, 2012 and March 31, 2013 met the inclusion criteria for participation. The exclusion criteria for the study were: i) Previous thyroid or parathyroid surgery; ii) previous neck surgery; iii) previous neck irradiation; iv) concomitant surgery for hyperparathyroidism; and v) surgery for locoregional recurrence.

### Surgery

Total thyroidectomy (TT) and CND were conducted by one of three surgeons during the study period, all using a similar technique. The study period was selected to maximize homogeneity of the surgical technique such that 70% of CNDs were performed by a single surgeon. An ultrasonic scalpel (Harmonic Focus; Johnson & Johnson, New Brunswick, NJ, USA) was used in cases of hemostasis. Routinely, recurrent laryngeal nerves were identified and exposed until their insertion in the larynx and parathyroid glands were identified and preserved. When devascularization or incidental removal of the parathyroid glands was suspected, a muscular autoimplantation procedure followed. Boundaries of the CND (level VI) were as decided according to the American Thyroid Association classification (8,9). Lymph nodes in this compartment included the pretracheal, paratracheal, prelaryngeal (Delphian) and perithyroidal nodes, including the lymph nodes medial and lateral to the recurrent laryngeal nerves. The superior boundary was defined as the cricoid cartilage, the inferior boundary was the innominate artery and the lateral boundaries were the common carotid arteries. Central neck dissection was performed as either an ipsilateral dissection, on the same side as the primary tumor, or as a bilateral dissection. The pretracheal lymph nodes were included with the ipsilateral CND. In general, bilateral CND was considered for patients with pre-operative or intra-operative evidence of ipsilateral central compartment adenopathy, contralateral central neck adenopathy or bilateral disease in the thyroid.

Following surgery, frequencies and patterns of CND metastases were analyzed with respect to patient characteristics, age and gender, and pathological variables, consisting of tumor size, histological type and primary tumor location (extra-thyroid invasion or not, multifocal or unifocal). For multiple primary lesions, the diameter of the largest dominant tumor was used in the analyses.

### Post-operative follow up

All clinical and pathological reports were reviewed. Routine follow-up at one, three and six months post-surgery, and every six months later, included neck ultrasonography and estimation of the serum thyroid stimulate hormone (TSH) level (reference range, 0.55–4.78 mU/l). Indirect laryngoscopy was performed prior to surgery and on the first day after the procedure. Vocal cord paresis that lasted for less than six months post-surgery was regarded as transient, but paresis that persisted for more than six months was regarded as permanent. The total serum calcium level (reference range, 2.08–2.60 mmol/l) was measured 24 h after surgery and medical treatment was initiated if the concentration was <2.08 mmol/l. Medication was started prophylactically in order to ensure that no patient developed hypocalcaemia symptoms. When the total serum calcium level was in the range of 1.8–2.08 mmol/l, calcium salts (1.5–3.0 g daily) were administered, and when the level was <1.8 mmol/l, calcium plus calcitriol (0.25–1.0 μg/day) was administered. The levels of serum calcium, serum phosphate (normal range, 0.81–1.45 mmol/l) and intact PTH (iPTH; chemiluminescence assay; normal range, 15–65 ng/l; detection limit, 6.0 ng/l) were determined prior to surgery, on the first day post-surgery and at every follow-up subsequent to the procedure. A serum calcium level of <2.08 mmol/l together with a subnormal serum iPTH level (<15 ng/l) was defined as transient hypoparathyroidism if the level was restored to a normal value within six months after the withdrawal of calcium therapy. Permanent hypoparathyroidism was regarded as persistent hypocalcaemia with a serum iPTH level <15 ng/l for more than six months after surgery, which required substitution with calcium, with or without calcitriol.

### Statistical analysis

Categorical data were compared using χ^2^ analysis (univariate analysis) and logistic regression analysis (multivariate analysis) using SPSS Statistics version 18.0 (SPSS, Inc., Chicago, IL, USA). P<0.05 was considered to indicate a statistically significant difference.

## Results

Between January 2012 and March 2013, 389 PTC patients met the inclusion criteria for the present study, 287 females and 102 males (female:male ratio, 2.82:1) with a mean age of 42.58±10.87 years. The patients were submitted for TT + CND (Table I). All the patients were successfully followed up until March 2014 (median follow-up, 18.5 months; range, 12.0–25.5 months). In 14/389 cases (3.60%), parathyroid tissue was identified in the final histopathological analysis. The incidence of surgical complications is reported in Table II. In three patients (0.77%), a neck hematoma that required surgical re-exploration was observed. During the follow-up, only one patient (1/389; 0.26%), a 22-year-old female, experienced disease recurrence in the residual thyroid gland and received an additional surgery 18 months after the initial procedure. None of the patients experienced further metastasis or tumor-associated mortality.

The mean tumor size was 0.71±0.35 cm (range, 0.1–2 cm) and microcarcinoma (tumor size, ≤1 cm) was diagnosed in 332 patients (85.35%). The fact that all the tumors included were ≤2.0 cm was incidental. The histotype was papillary carcinoma in all patients. A total of 90 patients (21.14%) possessed multifocal tumors, 107 patients (27.51%) were found with tumor extrathyroid invasion and lymph node involvement was identified in 129 patients (33.16%) (Table I).

Clinicopathological factors affecting central nodal metastases in patients were assessed by univariate analysis and are summarized in Table III for patients with conventional PTC and in Table IV for patients with a tumor size of ≤1.0 cm. There was no significant difference between patients with central nodal metastases with respect to age [P=0.067; P-value for microcarcinoma (P_1_)=0.202] or tumor focality (P=0.289; P_1_=0.221). However, there were significant differences between patients with central nodal metastases with respect to gender (P=0.006; P_1_=0.007), tumor size (P=0.014; P_1_=0.001) and extrathyroid invasion (P=0.04; P_1_=0.039).

In the multivariable analysis, the male gender and tumor size were found to be significantly associated with central lymph node metastasis for all the patients in the study (Tables V and VI).

From the statistics, it was indicated that compared with females, males were more vulnerable to developing positive lymph node involvement in the present study. The incidence of central lymph node metastasis appeared to be higher with a larger tumor size. The correlation coefficient between tumor size and central lymph node metastasis was 0.228, which was calculated using a two-tailed Pearson product-moment correlation (P<0.01).

## Discussion

Differentiated PTC exhibits a high propensity to spread to regional lymph nodes. As aforementioned, the reported incidence of clinically positive lymph nodes ranges between 20 and 50% (2), and was 33.2% in the present study. A higher proportion (80–90%) of patients exhibit subclinical lymph node metastases (micrometastases) at the time of surgical intervention (10–12). Despite the high incidence, lymph node metastases are not considered prognostic for poor survival in patients with well-differentiated PTC (13). Therefore, treatment of the cervical lymph nodes in well-differentiated PTC remains controversial. The primary argument for performing CND in the treatment of well-differentiated PTC is to more accurately stage the tumor. Accurate staging allows for improved risk stratification and the more rational application of levothyroxine suppression and adjuvant therapy, such as iodine ^131^I ablation (2–4). The presence or absence of pathological lymph nodes in neck dissection specimens has been reported to correlate with the incidence of disease recurrence. Elective CND may aid in the prevention of local recurrences in the central compartment where re-operation can be challenging (2–4). Therefore, there are proponents and opponents of elective CND. Certain PTC lesions (~25%), particularly in the older patient population (≥45 years old), concentrate radioactive iodine poorly (14–16). In these cases, radioactive iodine treatment may not adequately treat residual nodal micrometastasis. Thus, it is likely that elective CND is most beneficial at the time of initial surgery for selected high-risk patients.

**Figure 1:**
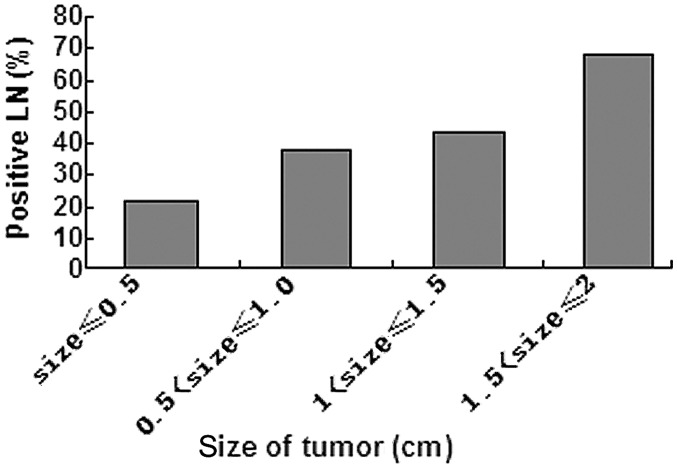
Different rate of central node metastasis in varying tumor size groups. The rates were 21.5, 37.8, 43.8, 67.8%, from left to right accordingly. LN, lymph node.

Determining the patients that are high risk prior to surgery remains difficult. The present study aimed to identify factors associated with central neck compartment nodal metastases as an initial step toward defining the patients that are most likely to benefit from elective CND. Malignant lymph nodes were found to occur with high frequency in male patients with a larger tumor size or extrathyroid invasion. Overall, from the statistical analysis, tumor size was found to be an extremely significant risk factor of central lymph node involvement in PTC. There was a significant difference (P<0.05) in the incidence of central lymph node metastasis between the four groups (Fig. 1) and the larger the tumor size, the higher the incidence. Only when the tumor size was ≤0.5 cm, was the incidence of lymph node involvement <30%.

Post-operative complications in the present study were comparative to those of other studies and to TT alone without CND (17–20). None of the patients underwent permanent hypoparathyroidism or permanent vocal cord paralysis. During the follow-up period, only one patient experienced recurrence. It appeared that CND did not increase the chance of the loco-regional recurrence. The safety of the surgery may partly be due to the high volume of surgeries and the experience of the surgeons at the Department of General Surgery at The Second Affiliated Hospital of Zhejiang University School of Medicine, with >1,000 cases treated per year. Therefore, the present conclusion is that it is advisable for male PTC patients (cN_0_) with a tumor size of ≥0.5 cm to receive prophylactic CND, particularly when performed by a surgeon that treats a high volume of PTC cases.

There are certain limitations to the present study. First, all the procedures were conducted by three surgeons. Although they were all surgeons who treat high volumes of PTC cases and used a similar technique, there are differences between them with regard to the extent of CND and the safety of the surgery. Second, the follow-up period was not long enough. Complications, particularly the recurrence rate or mortality rate due to PTC may change over time. The third limitation may also have been an advantage for the present study, as the tumor size was refined to 2.0 cm. If possible, an increased number of patients with larger tumor sizes should be included in the future. Further observation and study are required to better define the risk factors of central lymph node metastasis and the safety of CND in cN_0_ PTC.

## Figures and Tables

**Table I:** Demographic and pathological data of 389 papilliary thyroid carcinoma patients.

Feature	Value	Percentage
Patients		
Male	102	26.2
Female	287	73.8
Mean age, years	42.58±10.87^a^	
History		
Papillary cancer	389	100.0
Tumor		
Mean size	0.71±0.35^a^	
≤1 cm	332	85.4
>1 cm	57	14.7
Unique	299	76.9
Multifocal	90	23.1
Extrathyroid invasion	107	27.5
Positive LN	129	33.2

a Mean value ± standard deviation.

LN, lymph node.

**Table II:** Complications in 389 papillary thyroid carcinoma patients.

Complication	No. of patients	%
Transient hypothyroidism	48	12.34
Permanent hypothyroidim	0	0.00
Parathyroid tissue in the specimen	14	3.60
Transient unilateral vocal cord paralysis	16	4.11
Permanent unilateral vocal cord paralysis	0	0.00
Bilateral vocal cord paralysis	0	0.00
Neck hematoma	3	0.77

**Table III:** Clinicopathological factors affecting central nodal metastases.

Characteristic	No. of patients	No. of LN-positive patients (%)	P-value
Age, years			
<45	228	84 (36.8)	
≥45	161	45 (28.0)	0.067
Gender			
Male	102	45 (44.1)	
Female	287	84 (29.3)	0.006
Tumor size, cm			
≤1	332	102 (30.7)	
>1	57	27 (47.4)	0.014
Extrathyroid invasion			
Yes	107	44 (41.1)	
No	282	85 (30.1)	0.040
Tumor focality			
Unique	299	95 (31.8)	
Multifocal	90	34 (37.8)	0.289

LN, lymph node.

**Table IV:** Clinicopathological factors affecting central nodal metastases in microcarcinoma patients (n=332).

Characteristic	No. of patients	No. of LN-positive patients, %	P_1_-value
Age, years			
<45	201	67 (33.3)	
≥45	131	35 (26.7)	0.202
Gender			
Male	85	36 (42.4)	
Female	247	66 (26.7)	0.007
Tumor size, cm			
≤0.5	144	31 (21.5)	
>0.5	188	71 (37.8)	0.001
Extrathyroid invasion			
Yes	80	32 (40.0)	
No	252	70 (27.8)	0.039
Tumor focality			
Unique	255	74 (29.0)	
Multifocal	77	28 (36.4)	0.221

LN, lymph node.

**Table V:** Multivariate logistic regression analysis of central lymph node involvement.

	Variables in the equation					
	
Risk factors	B	S.E.	Wald	df	Sig.	Exp(B)
Gender	0.650	0.246	7.016	1	0.008	1.916
Tumor size	1.319	0.337	15.288	1	0.000	3.738
Extrathyroid invasion	0.182	0.255	0.510	1	0.475	1.200
Constant	−1.907	0.279	46.848	1	0.000	0.149

B, coefficient of regression; S.E., standard error of B; Wald, statistical value (χ^2^) used for hypothesis testing of B; df, degrees of freedom; Sig., P-value; Exp(B), adjusted odds ratio.

**Table VI:** Multivariate logistic regression analysis of central lymph node involvement in microcarcinoma.

	Variables in the equation					
	
Risk factors	B	S.E.	Wald	df	Sig1.	Exp(B)
Gender	0.760	0.271	7.832	1	0.005	2.137
Tumor size, cm	1.958	0.572	11.708	1	0.001	7.086
Extrathyroid	0.235	0.292	0.648	1	0.421	1.265
Constant	−2.312	0.388	35.532	1	0.000	0.099

B, coefficient of regression; S.E., standard error of B; Wald, statistical value (χ^2^) used for hypothesis testing of B; df, degrees of freedom; Sig1., P-value; Exp(B), adjusted odds ratio.
